# Student protests in Serbia: change the country or change countries

**DOI:** 10.1016/j.lanepe.2025.101439

**Published:** 2025-08-28

**Authors:** Biljana Vučković, Milica Krčo

**Affiliations:** Faculty of Medicine, University of Novi Sad, Serbia

In recent months, Serbia has witnessed growing concern for democratic values, justice, and institutional functionality. Although the entire country has suffered, two of Serbia's most vital pillars—healthcare and education at all levels—have endured mounting pressures. Medical professionals and academic staff, traditionally among the country's most respected voices, increasingly report a climate of constraint in which decisions affecting public welfare and institutional independence are made without sufficient transparency or inclusive dialog.^1^ Leadership across all public sectors appears to be operating under a model that prioritizes loyalty and control over merit and autonomy. This alarming trend reveals a more profound issue: the very institutions meant to serve the public, particularly the healthcare system, are being repurposed and used as a weapon of authoritarianism, rather than a fundamental human right available to everyone, regardless of their political affiliation.^2^

As mentioned in the *Lancet Regional Health - Europe's* August Editorial, the reaction to these events was an entire generation, long silenced and underestimated, finally standing up.^3^ This student-led movement, anchored by university blockades, has a set of strikingly simple, yet profound demands.^4^ They insist that the tragic fall of the Novi Sad canopy, not be downplayed as an accident, but recognized as a direct consequence of negligence and a corrupt system, calling for full political and civil accountability. The students stand up for the fundamental right of citizens to protest peacefully, free from intimidation and legal persecution. Finally, a demand that highlights the student-led nature of the movement and its focus on the future, calls for a significant reinvestment in higher education. This is a crucial investment in the institutions that will shape the next generation, aiming to build a stronger nation and actively preventing a return to the failures of the current system. For a time, it seemed possible that these demands might be met. However, the control exerted over state institutions has since rendered this impossible, prompting the students' call for early parliamentary elections as the necessary next step.^5^

Our vision for the future is clear: we seek a return to a nation where ethical principles and professional protocols are held paramount over political expediency. We envision a future where professionals, including doctors, are empowered with the freedom to reject proposals that compromise their integrity. This principle, which lies at the heart of the current student movement, has now awakened a nation-wide effort, inspiring people to rise from years of lethargy and despair and to fearlessly fight for their goals.

It is imperative that this crucial movement is not reduced to a footnote in history, and so we ask our international peers to stand with us in defending the very values—integrity, autonomy, and justice—that are the bedrock of every democratic society (Panel 1).

Panel 1 **Students' demands.**

1. Publication of the complete documentation concerning the reconstruction of the Novi Sad Railway Station, which is currently inaccessible to the public.
2. Confirmation from the relevant authorities of the identity of all persons for whom there is reasonable suspicion that they physically assaulted students and professors, and we also demand the initiation of criminal proceedings against them. Furthermore, we demand the dismissal of these persons if it is shown that they hold public office.
3. The withdrawal of criminal charges against arrested and detained students during the protests, as well as the suspension of already initiated criminal proceedings.
4. An increase of 20% in the funding allocated to state universities.

## Contributors

Prof. dr Biljana Vučković and sixth year student of the Faculty of Medicine in Novi Sad, Milica Krčo, have conceptualized, researched, wrote and edited the manuscript.

## Declaration of interests

The authors have no competing interests to declare.
